# Evolution mechanism of industrial network in Yangtze River Delta region from the perspective of link prediction

**DOI:** 10.1371/journal.pone.0308544

**Published:** 2024-09-20

**Authors:** Yue Shen, Yixin Ren, Yiwen Zhang

**Affiliations:** 1 College of Economics and Management, Shandong University of Science and Technology, Qingdao, China; 2 College of Business, Nanjing Normal University, Nanjing, China; Australian Catholic University, AUSTRALIA

## Abstract

The Yangtze River Delta (YRD) is an important engine of national economic development and a leading region in international competition. As economic exchanges and resource flows in the YRD region become closer, the inter-regional industrial linkages continue to grow, resulting in the formation of an industrial network structure characterized by a “complex network”. The strength of the links between industrial sectors and the value and significance of the existence of industries in the network change over time, thus causing the overall evolution of the industrial network in the YRD region. Based on the input-output tables of the YRD region in 2012 and 2017, this paper uses the prediction index of network structure similarity to construct the prediction model of industrial network link between the YRD regions, and calculates the possibility of future links between industries in the Yangtze River Delta region through comparative analysis and selection of the RWR index of random walk similarity with the best effect, and concludes that: (1) the homogeneity of industries among provinces and cities in the Yangtze River Delta region is relatively high, resulting in homogeneous competition; (2) the overall nature of the industrial layout of the YRD is not prominent, and the depth and intensity of cross-regional industrial cooperation are lacking. On the basis of analysis and research, the countermeasures and suggestions for effectively realizing industrial integration are put forward from the macro level of the government and the meso level of the industry, so as to achieve a more complete industrial network in the YRD region and a more extensive length and width of the cross-regional industrial chain.

## Introduction

The Yangtze River Delta (YRD) is an important engine for the country’s economic development and a pioneering region to participate in international competition, both in terms of economic development and industrial transformation, which puts forward an urgent need for the coordinated development of the four regions. In 2017, the report of the 19th National Congress of the Communist Party of China (CPC) upgraded the coordinated development of the region as one of the seven national strategies, and made a specific deployment of coordinated development strategies in the region, putting forward the coordinated development of Beijing-Tianjin-Hebei, the development of the Yangtze River Economic Belt, the eastern regional Optimization and Development, Eastern Revitalization, Central Rise, and Western Development Strategies. In 2019, the YRD region has gone through the process from the establishment of the Shanghai Economic Zone to the rise of the YRD Regional Integration as a national strategy. Globally, it is a general trend that city clusters have become an important carrier for the transfer of the world’s economic centers, determining the future pattern of the world’s political and economic development. Domestically, it is a major strategy determined by the central government, and urban agglomeration is also the “main engine” leading China’s economic transformation and development, and the “main position” for innovation and development. Since the 18th Party Congress, the central government has attached great importance to the strategy of coordinated regional development. From its own point of view, it is an inherent requirement. The YR region is the intersection of China’s eastern coastal economic zone and the Yangtze River economic zone, and is an important industrial and agricultural production base in China. The development of the YRD can lead to the development of the Yangtze River and coastal areas, and radiate to the whole country, driving the economic development of the vast areas of China. As the YRD region has an important strategic geographic location, closely connected to the “One Belt and One Road” and adjacent to the Yangtze River Economic Belt, it is the key hub of China’s opening up to the outside world, shouldering the dual tasks of driving the development of the central and western regions internally and participating in global cooperation and competition externally, and it is necessary to break through integration innovations and better serve the overall situation of the country’s development. It is necessary to make innovative breakthroughs through integration to better serve the overall situation of national development. However, the industrial synergy effect of the YRD has not been fully utilized, and Anhui Province has not yet been integrated into the YRD region. Now that the integration of the YRD has been given high priority, will it be possible to truly integrate the four regions together?

With the closer economic exchanges and resource flows in the YRD region, the inter-regional industrial linkages have been increasing, and the inter-industry linkages are no longer confined to the intra-regional linkages, but the inter-regional linkages have also formed a complex network structure of industries with the characteristics of “complex networks”. The strength of the links between industrial sectors and the value and significance of the existence of industries in the network have changed over time, thus causing the overall evolution of the industrial network in the YRD region. At present, the coordinated development of industries in the YRD region is complementary, and the system of industrial division of labor and collaboration among the four places has not yet taken shape, and the four places have different positioning within each other. At the same time, there are different degrees of industrial structure similarity, irrational resource allocation and vicious regional competition among the three provinces and one city. The industrial pattern of the YRD region needs to improve the division of labor and coordination, with the core industry as the leader, relying on the role of radiation, to achieve the coordinated development of the entire YRD regional industrial exchanges.

## Literature review

Many systems in real world domains, whether physical or abstract, can be described as complex networks. In these networks, nodes represent individuals or actors, and connected edges represent various relationships or interactions between them. These systems are inherently dynamic and evolve over time as individuals or actors arrive and/or leave simultaneously and links between individuals or actors in the network form, strengthen, weaken, and disappear simultaneously. Link prediction, as an important mechanism for network evolution, has received widespread attention as soon as it was proposed and has been practically applied in many fields [1–4]. Currently, link prediction in networks refers to predicting the possibility of establishing links between nodes in the network that have not yet established links. For many real networks, the purpose of network evolution is to extend the network topology to ensure long-term growth and proper operation. Therefore, the study of link prediction is necessary to improve the reliability of network evolution. Since it is difficult to obtain effective information, scholars have proposed a link prediction method based on the similarity of network structure to address the information problems that exist. At present, the research content of the regional industrial network in the YRD is mainly based on the construction of regional industrial network and the analysis of its network structure characteristics, ignoring the research on the evolution of industrial network.

Domestic scholars’ research on the industrial network in the YRD region mainly focuses on the formation and construction of the network, and the measurement of the characteristics of the network results. For example, Mao Xiyan et al. combined input-output analysis and complex network analysis to identify the path characteristics of cross-regional linkages of industries, and then analyzed the network structure characteristics of cross-regional linkages of polluting and non-polluting industries in the Yangtze River Economic Belt [5]. From the perspective of complex network analysis, Wu Hui et al. used the data processing technique of programmed rooting theory to process the data, and took the biomedical industry as the research object to construct the framework of four contents, three subjects, two bridges, and three modes of collaborative innovation integration of industries in the YRD region, and to explore the processes and paths of the integration of the YRD region [6]. Ma Jing et al. took the biomedical industry in the YRD region as the research object, chose the data of collaborative patent applications from 2001 to 2018 to construct the spatial structure of the innovation network among cities in the YRD region for exploration, and analyzed the influence mechanism of the network structure with the help of the panel negative binomial fixed-effects model [7]. Sun et al. used entropy weight to improve the CRITIC method for the YRD city cluster industrial ecological efficacy in and out of the measurement, and established a directed network to analyze the synergistic effect of industrial ecology in the YRD city cluster [8]. At present, the research content of the regional industrial network in the YRD is mainly based on the construction of regional industrial network and the analysis of its network structure characteristics, ignoring the research on the evolution of industrial network.

Domestic and foreign scholars’ research on link prediction for network structure similarity mainly focuses on improving existing link prediction algorithms and verifying the effectiveness of link prediction algorithms. For example, Xu and Yin (2017) effectively combined CN metrics and RA metrics to improve the ability of prediction accuracy through known interactions between nodes [9]. Sun et al. utilized CN metrics to build link prediction models with community structure information to improve the prediction performance [10]. Muniz et al. proposed to combine the global similarity degree index and node attribute combination to improve link prediction performance [11]. Zhang Ruofan et al. took the industry-university-research cooperation in Guangdong, Hong Kong and Macao Greater Bay Area as the research object, constructed the application network of industry-university-research cooperation from 2016 to 2020, and utilized AA indicators to predict the possibility of patent technology cooperation opportunities in Guangdong, Hong Kong and Macao Greater Bay Area [12]. Ding Jingda and Guo Jie introduced weights in the author cooperation network and used a combination of author research content similarity degree and cooperation network structure similarity to predict the likelihood of potential author partnerships [13]. Li Bing et al. established a firm-patent heterogeneity network and applied a link prediction algorithm with random wandering SimRank metrics to predict and analyze the firm’s potential technology partners and competitors [14]. Tan Zihao proposed a link prediction method for AA metrics by adding the decay of time parameters to calculate the similarity between electric cabs and charging stations [15]. Chang Xiaomeng proposed a topology-based temporal link prediction algorithm to improve the accuracy of link prediction [16]. Yingjie Liu et al. proposed a link prediction algorithm combining neighbor influence and resource allocation to explore the influence of common neighbors and secondary nodes on predicting the possibility of the existence of a connecting edge between two nodes [17]. Yang et al. took the top 10 full-service airlines in the world as the research object, used five endogenous attribute similarity indexes and three exogenous attribute similarity indexes as well as coupled similarity indexes to calculate the AUC value respectively, selected the similarity indexes with the highest AUC value to perform the route prediction and validation, and constructed a link prediction-based dynamic weighted BBV for the improvement of the route network of the full-service airlines evolution model [18]. At present, scholars at home and abroad focus on the research of link prediction theory with similar network structure, and there is a relative lack of applied research on link prediction.

In summary, it can be seen that the current research on industrial networks in the YRD region mainly focuses on the construction of networks and the analysis of network structural features, and there are few studies on the evolution of industrial networks in the YRD. Meanwhile, the current literature on link prediction of network structure similarity mainly focuses on the research related to link prediction algorithms, and the literature on link prediction application is less, especially the literature on link prediction of industrial networks is very little. Based on the YRD regional industrial network, this paper chooses a scientific and reasonable link prediction method of network structure similarity to predict the possibility of future linkage between any two industries in the YRD regional industrial network, so as to analyze the trend of YRD regional industrial network evolution. Since the national and provincial input-output tables are compiled once every five years and there is a lag in the compilation and release of the input-output tables, predicting the possibility of inter-industry links in the network can be a good way to foretell the development of the industrial network paths, which will provide a theoretical basis for the early intervention, adjustment of the regional industrial system and optimization of the regional development.

## Theoretical framework of link prediction

In recent years, with the rapid development of network science, its theoretical results have built a good research platform for link prediction, which makes the study of link prediction closely linked to the structure and evolution of networks. In the meantime, link prediction can fully explore all kinds of information in the network system, provide data support for network evolution research, and help to understand the complex network evolution mechanism. However, the link prediction method is still blank in the field of econophysics that studies regional industrial economic problems, especially in the field of industrial complex network application research. Therefore, this paper will use the link prediction method to study the industrial network evolution problems in regional economics on the basis of industrial complex networks, enriching the analytical tools of complex networks in econophysics.

### Description of the problem

A network *G*(*V*, *L*) is composed of a finite non-empty set of nodes *V* and a finite set of edges between unordered nodes *L*. |*V*| denotes the number of nodes in the network and |*L*| denotes the number of edges between nodes in the network. The theoretical maximum value of the number of possible node edges in the network is *U* = *N* × (*N* − 1)/2. Then *U* − *L* represents the unobserved edges in the network, which includes edges that already exist but are currently unknown and edges that currently do not exist but may arise in the future. Thus, link prediction aims to identify as many connecting edges from *U* − *L* as possible that are unknown or may exist in the future.

Assuming an undirected network *G*(*V*, *L*), a fraction value *s*_*xy*_ is computed for each pair of node pairs *v*_*x*_, *v*_*y*_ that are not connected with edges, using any of the link prediction methods that are similar in network structure. Based on this score value *s*_*xy*_, it can be determined whether the unconnected pairs of nodes will have a connecting edge in the future, which is positively correlated with the probability of connection between the two nodes. All unconnected node pairs are sorted in descending order of the fractional value *s*_*xy*_. The node pair at the top of the list has the greatest chance that a connection may occur [19].

### Data set segmentation

For a given network *G*(*V*, *L*), in order to satisfy the need to evaluate the accuracy of the prediction method, it is necessary to divide the set of connected edges *L* that already exists in the network species into two sets i.e., the training set *L*^*T*^ and the test set *L*^*P*^. For computing the score value, only the information in the training set is used, with *L* = *L*^*T*^ ⋃ *L*^*P*^, and *L*^*T*^ ⋂ *L*^*P*^ = Φ. Edges belonging to *U* but not to *L* are defined as unobserved edges ℜ and edges belonging to *U* but not to *L*^*T*^ are defined as unknown edges *L*^*U*^. There are various methods for dividing the training and test sets, such as random sampling, item-by-item traversal, k-fold cross-test, etc., while random sampling is the most commonly used method. Therefore, in this paper, we choose the random sampling method, set the proportion of test set selection as *p*(*p*∈(0,1)), then the random sampling method will randomly select *L* edges from *μL* known edges as the test set. The notations and symbols employed in this paper are shown in Table 1.

**Table 1:** Notations employed in this article and their corresponding descriptions.

Notations	Description
*G*(*V*, *L*)	*G* is an undirected graph, *V* is the set of nodes and *L* is the set of edges in *G*, respectively.
*n* = |***V***|	The number of nodes in *G*.
*m* = |*L***|**	The number of edges in *G*.
*V* = {*v*_1_, *v*_2_, …, *v*_*n*_}	*V* stands for the nodes set of *G*, and *v*_*i*_ represents the i-th node.
*L* = {*l*_1_, *l*_2_, …, *l*_*m*_}	*L* stands for the observed edges set of *G*, and *l*_*i*_ represents the i-th edge.
*e*_*ij*_	The edge between node *v*_*i*_ and *v*_*j*_.
ℜ=U−L	ℜ represents the unobserved edges in *G*.
*L*^*U*^ = *U* − *L*^*T*^	*L*_*U*_ represents the unknown edges in *G*.

### Link prediction algorithm

The link prediction algorithm based on network structure similarity is not only able to analyze the local or global structural characteristics of the network, but also able to take into account the external attributes of the nodes, and the advantage of this network structure similarity link prediction model lies in the fact that the higher the similarity scores between the nodes, the higher the probability of connectivity, and the easier it is to connect. Network structure similarity indexes can be roughly classified into three types: similarity indexes for local information, similarity indexesor paths, and similarity indexes for random wandering.

#### Similarity indexes based on localized information

Similarity indexes based on local information are similarity indexes calculated using only node local information (e.g., node’s degree and nearest neighbor), and this category includes 10 types of indexes such as CN index, AA index, RA index, and so on.

*Common Neighborhood Index (CN)*. Assuming that if two nodes in a network have multiple common neighbor nodes, there may be a connecting edge between these two nodes [20]. The CN index of nodes and nodes in the network can be expressed as:

$$S_{\text{ij}}=\left|\Gamma \left(v_{i}\right)\cap \Gamma \left(v_{j}\right)\right|$$ (1)

Where Γ(*v*_*i*_), Γ(*v*_*j*_) denote the set of neighbors of node *v*_*i*_ and node *v*_*j*_ respectively.

On the basis of common neighbors need to consider the effect of node degree at both ends, from different perspectives and in different ways will produce six similarity indexes, as shown in Table 2.

**Table 2:** Six similarity indexes based on local information.

Index	Formulas	Index	Formulas
Salton [20]	$S_{ij}=\frac{\left|\Gamma \left(v_{i}\right)\cap \Gamma \left(v_{j}\right)\right|}{\sqrt{k\left(v_{i}\right)\times k\left(v_{j}\right)}}$	HPI [21]	$S_{ij}=\frac{\left|\Gamma \left(v_{i}\right)\cap \Gamma \left(v_{j}\right)\right|}{\text{min}\left\{k\left(v_{i}\right),k\left(v_{j}\right)\right\}}$
Jaccard [20]	$S_{ij}=\frac{\left|\Gamma \left(v_{i}\right)\cap \Gamma \left(v_{j}\right)\right|}{\left|\Gamma \left(v_{i}\right)\cup \Gamma \left(v_{j}\right)\right|}$	HDI [22]	$S_{ij}=\frac{\left|\Gamma \left(v_{i}\right)\cap \Gamma \left(v_{j}\right)\right|}{\text{max}\left\{k\left(v_{i}\right),k\left(v_{j}\right)\right\}}$
Sørenson [20]	$S_{ij}=\frac{\left|\Gamma \left(v_{i}\right)\cap \Gamma \left(v_{j}\right)\right|}{\left|k\left(v_{i}\right)\cup k\left(v_{j}\right)\right|}$	LHN-I [23]	$S_{ij}=\frac{\left|\Gamma \left(v_{i}\right)\cap \Gamma \left(v_{j}\right)\right|}{k\left(v_{i}\right)\times k\left(v_{j}\right)}$

*Adamic Adar Index (AA)*. The core idea is that the contribution of the common neighbor node with small degree is greater than that of the common neighbor node with large degree [24]. The AA index uses the weights of the common neighbor nodes of two nodes as the similarity of these two nodes, which is given by the formula:

$$S_{ij}=\sum_{z\in \Gamma \left(v_{i}\right)\cap \Gamma \left(v_{j}\right)}\frac{1}{\text{log}\;k\left(z\right)}$$ (2)

*Resource allocation Index (RA)*. Based on the idea of resource transfer on a network, there are two nodes *v*_*i*_ and *v*_*j*_ that do not have consecutive edges on the network, and node *v*_*i*_ can transfer some resources to node *v*_*j*_. Their common neighbors play the role of transfer [23, 25]. If a unit of resource is passed from node *v*_*i*_ to node *v*_*j*_ and is equally distributed to the neighbors of node *v*_*i*_, the similarity between node *v*_*i*_ and node *v*_*j*_ can be defined as the number of resources node *v*_*j*_ receives from node *v*_*i*_, and the number of resources node *v*_*j*_ receives from node *v*_*i*_, which is given by the formula:

$$S_{ij}=\sum_{z\in \Gamma \left(v_{i}\right)\cap \Gamma \left(v_{j}\right)}\frac{1}{k_{v_{i}}}$$ (3)

*Preferential Attachment Index (PA)*. In the network model, at each step a link is first removed and then a link is added. The probability that such a new link connects node *v*_*i*_ and node *v*_*j*_ is proportional to the product of the degrees of the two nodes, and motivated by this mechanism, the corresponding similarity is defined as:

$$S_{ij}=k\left(v_{i}\right)\times k\left(v_{j}\right)$$ (4)

#### Path-based similarity indexes

Path-based similarity indexes are predicted using information about the number of paths of different lengths between nodes, which include: the local path index (LP), the Katz index and the LHN-II index.

*Local Path Index (LP)*. The prediction is performed using the linear superposition of the number of second-order and third-order paths between the nodes [17], and its expression is:

$$S=A^{2}+\alpha A^{3}$$ (5)

*Katz Index*. The prediction is made using the linear superposition of the number of paths of all lengths between the nodes [23], and its expression is:

$$S=\beta A+\beta^{2}A^{2}+\beta^{3}A^{3}+\cdots +\beta^{n}A^{n}={\left(I-\beta A\right)}^{-1}-I$$ (6)

*LHN-II Index*. The basic idea is based on general equivalence, assuming that in a network where node *v*_*i*_ and node *v*_*j*_ connected by nodes are similar to each other, then these two nodes are also similar to each other [23], the LHN-II metric can be defined as:

$$S=2M\lambda_{1}D^{-1}\left(I-\frac{\phi A}{\lambda_{1}}\right)D^{-1}$$ (7)

#### Similarity indexes based on random walks

Similarity indexes based on random wandering are used for link prediction by simulating the process of random wandering of particles, assuming the condition that the lower the average number of steps required for node *v*_*i*_ to randomly wander to reach node *v*_*j*_ the higher the similarity between these two nodes. The similarity indexes of random wandering include local random wandering and global random wandering. Among them, the local random walk only considers the random walk process with a limited number of steps, which does not make full use of the network structure information, and the prediction results are relatively poor; although the global random walk utilizes the global information of the network, the computation process is more complicated, and it is not applicable to larger networks. Local random walk includes local random walk indicator and SRW index (superimposed local random walk index). Global random walk includes ACT index (average commuting time), Cos+ index (cosine similarity based on random walk), RWR index (random walk index with restart) and SimR index. In this paper, the ACT index, Cos+ index, and RWR index are chosen as the similarity indexes of randomized wandering.

*ACT Index*. Assuming that A is the average number of steps a random particle needs to take from node *v*_*i*_ to node *v*_*j*_ [26], then the expression for the average commuting time at node *v*_*i*_ and node *v*_*j*_:

$$n\left(v_{i},v_{j}\right)=m\left(v_{i},v_{j}\right)+m\left(v_{j},v_{i}\right)$$ (8)

Its value can be obtained by finding the pseudo-inverse *E*^+^ of the Laplace matrix *E* of this network, i.e.

$$n\left(v_{i},v_{j}\right)=M\left(e_{ii}^{+}+e_{jj}^{+}-2e_{ij}^{+}\right)$$ (9)

Where $e_{xy}^{+}$ denotes the element at the corresponding position in the matrix *E*^+^. If the average commuting time of two nodes is smaller, then the two nodes are approximately close. As a result, the similarity of ACT is

$$S_{ij}=\frac{1}{e_{ii}^{+}+e_{jj}^{+}-2e_{ij}^{+}}$$ (10)

*Cosine Similarity Based on Random Walks (Cos+)*. The inner product of the vector *i* and the vector *j*, $e_{ij}^{+}=w_{i}^{T}w_{j}$ can be represented as an element $e_{ij}^{+}$ of the pseudo-inverse matrix *E*^+^, which must be in the space expanded by *w*_*i*_ = Λ^1/2^*U*^*T*^*ε*_*i*_. *U*^*T*^ is a standard orthogonal matrix obtained by arranging *E*^+^ in ascending order according to the corresponding eigen roots, and Λ is the diagonal element whose diagonal elements are the eigen roots [27]. The matrix transpose uses the superscripts *T*, *ε*_*i*_ denotes a one-dimensional vector with only the i-th element being 1 and the others being 0. This defines the cosine similarity as:

$$S_{ij}=\text{cos}{\left(v_{i},v_{j}\right)}^{+}=\frac{e_{ij}^{+}}{\sqrt{e_{ii}^{+}e_{jj}^{+}}}$$ (11)

*Stochastic Wanderer with Restarts (RWR)*. The application of this metric is based on certain assumptions: whenever a random wandering particle travels each step, it returns to its initial position with a particular probability [28]. The return probability of a particle is denoted by 1 − *c*. The probability transfer matrix is *p*, and its element *P*_*ij*_ = *a*_*ij*_/*k*_*i*_ denotes the probability that the particle at node *v*_*i*_ will walk to node v_*j*_ next. A particle is at node *v*_*i*_ at the initial moment, then the probability vector of the particle reaching each node of the network at time *t* + 1 is:

$$q_{i}\left(t+1\right)=cP^{T}q_{i}\left(t\right)+\left(1-c\right)e_{i}$$ (12)

Where *e*_*i*_ denotes the initial state and the steady state solution to the above equation is

$$q_{i}=\left(1-c\right){\left(I-cP^{T}\right)}^{-1}e_{i}$$ (13)

Where the element *q*_*ij*_ is the probability that a particle departing from node *v*_*i*_ will eventually travel to node *v*_*j*_. The resulting RWR similarity is:

$$S_{ij}=q_{ij}+q_{ji}$$ (14)

### Precision evaluation indicators

The current metrics for measuring the accuracy of link prediction algorithms are AUC proposed by Hanely J A et al. [29], Precision proposed by Herlocker J L et al. [30], and Ranking Score proposed by Zhou T. et al. They have different emphasis on the measurement of prediction accuracy. Among them, AUC measures the accuracy of the algorithm as a whole, Precision only considers whether the top L edges are predicted accurately or not, while Ranking Score considers more about the ordering of the predicted edges [31].

#### AUC

It is the area under the ROC Curve (Receiver Operating characteristic Curve), which is based on the principle of calculating the likelihood magnitude of all unknown links by the link prediction algorithm, and then plotting the distribution of the likelihood of test links and nonexistent links, i.e., plotting the ROC curve [31]. Thereafter the area under the curve is calculated so as to evaluate the algorithm. The specific process is to randomly select an edge from the test set each time, and then randomly select a nonexistent edge, if the score value of the edge in the test set is higher than the score value of the nonexistent edge, then 1 point is added, and if the two scores are equal then 0.5 points are added. In *z* independent comparisons, there exist *z*′ times when the edge score value in the test set is greater than the score value of the non-existent edge, *z*″ the two score values are equal, then AUC is defined as:

$$AUC=\frac{z^{\prime }+0.5z^{\prime \prime }}{z}$$ (15)

The larger the AUC value, the better the prediction of the model.

#### Precision

It is defined as the proportion of the previous *L* predicted edges that are accurate [29]. If *M* predictions are accurate, that is, in order from greatest to least likely value of connection, M of the top L edges are in the test set, then Precision is defined as:

$$Precision=\frac{M}{L}$$ (16)

Obviously, the magnitude of the Precision value is related to the number of edges taken *L*. The range of Precision is [0,1] Assuming that *L* is constant, a larger Precision value indicates a higher accuracy of the prediction algorithm.

## Construction and analysis of industrial network in Yangtze River Delta region

The YRD regional industrial network is composed of 168 nodal industries in three provinces and one city, and its inter-nodal links indicate inter-industry supply relationships. In order to facilitate the study, the following assumptions are made while satisfying the construction conditions:

1. The study in this paper does not consider the type of industry, but only takes the industry as a node in the network.
2. If there is a mutual supply relationship between industries, there exists a connecting line between them without considering the directionality of the connecting edge.
3. Abstract the inter-industry cooperative relationship as an undirected network, ignore the upstream and downstream relationship between industries, and default the transfer information between industries is symmetric, so there is no assignment of value to the edge.

### Data sources

Since there is a lag in the process of compiling and releasing the input-output tables of the three provinces and cities in the YRD and the national input-output table, for the sake of data availability and caliber unity, the most recent input-output tables of the Shanghai, the Jiangsu Provence, the Zhejiang Provence, the Anhui Provence and the national input-output table are chosen to be the 2012 and 2017, so the required 2012 and 2017 inter-regional input-output tables of the YRD are compiled, and from this, we calculated the get the direct consumption coefficient matrix. On this basis, the Weaver Thomas Index (WI Index) is used for thresholding to obtain the required adjacency matrix *A*_2012_ and *A*_2017_.

$$A_{2012}={\left[\begin{array}{lllllllllllll} 0 & 0 & 0 & 0 & 0 & 1 & \cdots & 0 & 0 & 0 & 0 & 0 & 0\\ 0 & 0 & 0 & 0 & 0 & 0 & \cdots & 0 & 0 & 0 & 0 & 0 & 0\\ 0 & 0 & 0 & 0 & 0 & 0 & \cdots & 0 & 0 & 0 & 0 & 0 & 0\\ \vdots & \vdots & \vdots & \vdots & \vdots & \vdots & \cdots & \vdots & \vdots & \vdots & \vdots & \vdots & \vdots \\ 0 & 0 & 0 & 0 & 0 & 0 & \cdots & 0 & 0 & 0 & 0 & 0 & 0\\ 0 & 0 & 0 & 0 & 0 & 0 & \cdots & 0 & 0 & 0 & 0 & 0 & 1\\ 0 & 0 & 0 & 0 & 0 & 0 & \cdots & 0 & 0 & 0 & 0 & 0 & 0 \end{array}\right]}_{168\times 168}$$

$$A_{2017}={\left[\begin{array}{lllllllllllll} 0 & 0 & 0 & 0 & 0 & 1 & \cdots & 0 & 0 & 0 & 0 & 0 & 0\\ 0 & 0 & 0 & 0 & 0 & 0 & \cdots & 0 & 0 & 0 & 0 & 0 & 0\\ 0 & 0 & 0 & 0 & 0 & 0 & \cdots & 0 & 0 & 0 & 0 & 0 & 0\\ \vdots & \vdots & \vdots & \vdots & \vdots & \vdots & \cdots & \vdots & \vdots & \vdots & \vdots & \vdots & \vdots \\ 0 & 0 & 0 & 0 & 0 & 0 & \cdots & 0 & 0 & 0 & 0 & 0 & 0\\ 0 & 0 & 0 & 0 & 0 & 0 & \cdots & 0 & 0 & 0 & 0 & 0 & 0\\ 0 & 0 & 0 & 0 & 0 & 0 & \cdots & 0 & 0 & 0 & 0 & 0 & 0 \end{array}\right]}_{168\times 168}$$

### Network structure analysis

#### Network macrostructure analysis

**Fig 1:**
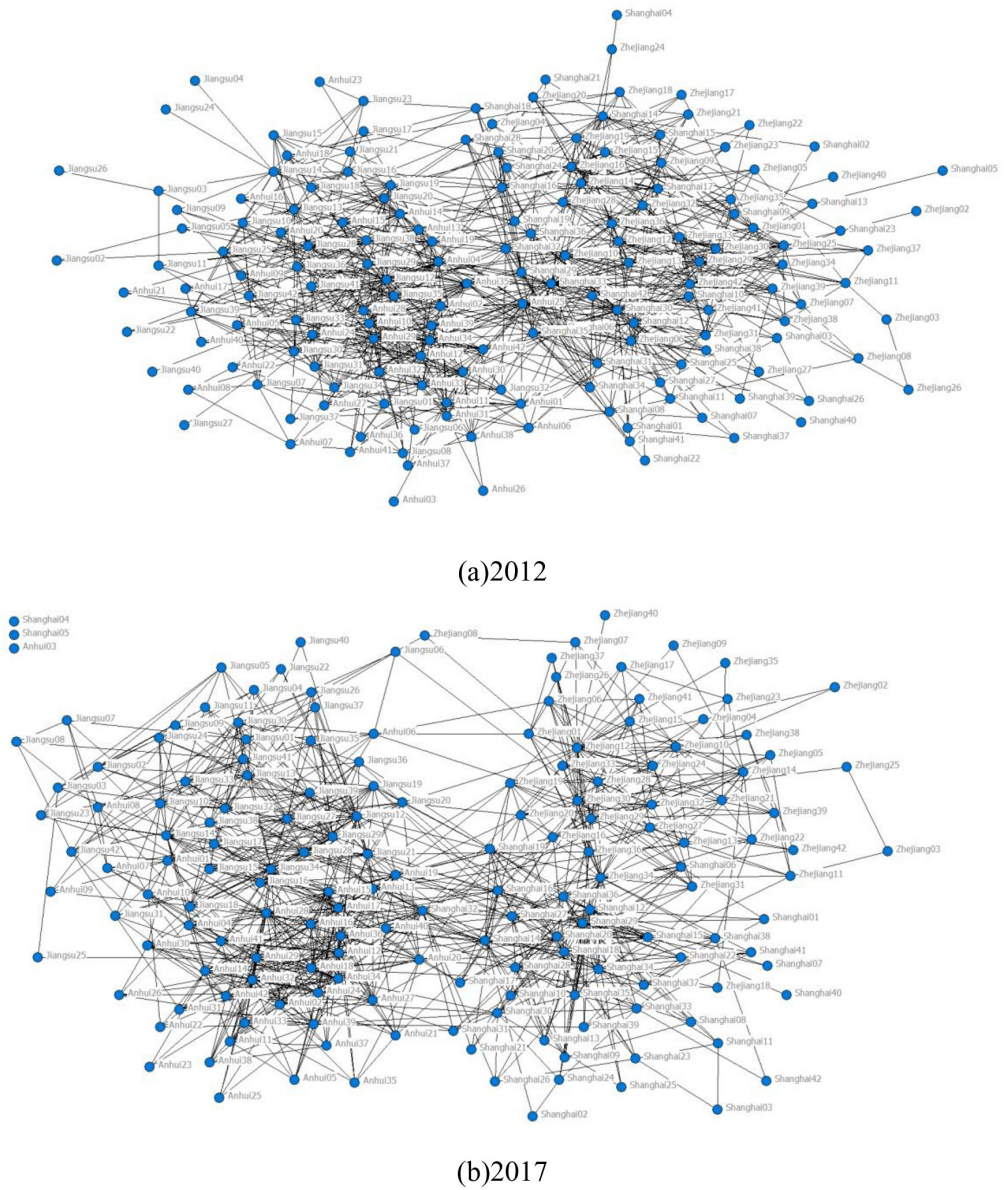
Industrial network map of the Yangtze River Delta region.

The adjacency matrix *A*_2012_ and *A*_2017_ are imported into the UCINET software, and the structure of the 2012 and 2017 YRD interregional industrial network is plotted in Fig 1.

By analyzing the macro-structural indicators of the YRD regional industrial network (Table 3), it is concluded that (1) the YRD regional industrial network has a low level of average path length and a high level of clustering coefficients, and exhibits an obvious small-world phenomenon; and (2) the YRD regional industrial network as a whole is relatively sparse, and there is not a close cooperative relationship between industries in the three provinces and one city.

**Table 3:** Macrostructural indicators of industrial networks in the Yangtze River Delta region.

	Number of nodes	Number of edges	Network density	Clustering coefficient	Average path length
**2012**	168	902	0.064	0.348	2.615
**2017**	168	923	0.066	0.361	2.634

#### Centrality analysis

Centrality refers to the degree of the node’s central position in the industrial network of the YRD region. A high degree of centrality indicates that a highly cooperative industry is at the core of the network or in an important position to connect two weakly connected groups. Exploring the centrality can reveal the industry’s influence on other industries and its ability to control the flow of information in the network. Analysis from the degree centrality and intermediate centrality: degree centrality is a measure of the centrality of a point, if a point has more direct connections with other points, then the centrality is larger; intermediate centrality is a measure of a point’s “intermediary” role in the network, the greater the intermediate centrality proves that the point is in the path of many other points, it may have a greater centrality, and it may have a greater influence on the network. The greater the meso-centrality, the greater the ability to control the interactions between other nodes.

From the analysis of centrality in Tables 4 and 5 below, it can be seen that Anhui Province accounted for the largest share of the top twenty centrality in 2012 and 2017. In 2017, the centrality and intermediate centrality rankings of Jiangsu Province’s “chemical products” rose rapidly, and it played a larger role as an intermediary in the network, and had a stronger cooperative relationship with other nodes. The reason for this is mainly that since the “13th Five-Year Plan”, the construction of chemical parks in Jiangsu Province has set off another climax and entered a new stage of improving quality and efficiency.

**Table 4:** Top 20 industries in the centrality of industrial networks in the Yangtze River Delta region.

2012		2017	
Provinces	Industry	Provinces	Industry
Shanghai	Financial	Jiangsu	Chemical products
Shanghai	Transportation, storage and postal services	Shanghai	Information transmission, software and information technology services
Anhui	Production and supply of electricity and heat	Anhui	Constructions
Anhui	Metal Ore Mining Products	Zhejiang	Real estates
Anhui	Wholesale and retail	Jiangsu	Real estates
Jiangsu	Chemical products	Shanghai	Chemical products
Zhejiang	Chemical products	Shanghai	Wholesale and retail
Anhui	Leasing and business services	Anhui	Wholesale and retail
Anhui	Metal smelting and rolling products	Zhejiang	Chemical products
Shanghai	Chemical products	Anhui	Metalwork
Zhejiang	Paper, printing and educational and sporting goods	Anhui	Chemical products
Zhejiang	Financial	Anhui	Electrical machinery and equipment
Shanghai	Food and tobacco	Jiangsu	Wholesale and retail
Zhejiang	Metal smelting and rolling products	Anhui	Information transmission, software and information technology services
Anhui	Chemical products	Anhui	Real estates
Anhui	Transportation, storage and postal services	Anhui	Specialized equipment
Jiangsu	Constructions	Jiangsu	Metal smelting and rolling products
Anhui	Real estates	Jiangsu	Constructions
Shanghai	Metal smelting and rolling products	Shanghai	Metal smelting and rolling products
Anhui	Electrical machinery and equipment	Shanghai	General equipment

**Table 5:** Top 20 industries in the betweeness centrality of industrial networks in the Yangtze River Delta region.

2012		2017	
Provinces	Industry	Provinces	Industry
Shanghai	Transportation, storage and postal services	Jiangsu	Chemical products
Shanghai	Financial	Shanghai	Information transmission, software and information technology services
Jiangsu	Chemical products	Zhejiang	Chemical products
Anhui	Metal Ore Mining Products	Shanghai	Chemical products
Anhui	Production and supply of electricity and heat	Zhejiang	Real estates
Zhejiang	Chemical products	Shanghai	Wholesale and retail
Shanghai	Chemical products	Jiangsu	Wholesale and retail
Anhui	Metal smelting and rolling products	Zhejiang	Transportation, storage and postal services
Anhui	Leasing and business services	Anhui	Constructions
Shanghai	Metal smelting and rolling products	Jiangsu	Real estates
Anhui	Wholesale and retail	Anhui	Metalwork
Zhejiang	Metal smelting and rolling products	Anhui	Chemical products
Jiangsu	Metal smelting and rolling products	Anhui	Wholesale and retail
Shanghai	Food and tobacco	Shanghai	Metal smelting and rolling products
Zhejiang	Paper, printing and educational and sporting goods	Anhui	Electrical machinery and equipment
Shanghai	Scientific research and technical services	Zhejiang	Wholesale and retail
Anhui	Transportation, storage and postal services	Shanghai	General equipment
Jiangsu	Leasing and business services	Anhui	Non-metallic mineral products
Jiangsu	Constructions	Jiangsu	Constructions
Anhui	Chemical products	Zhejiang	Constructions

## Simulation model for predicting industrial network links in the Yangtze River Delta region

This part focuses on the linking of the YRD regional industrial network and the regional mapping in order to explore the inter-industry and inter-regional cooperation opportunities. According to the meaning of link prediction, it is necessary to eliminate the independent nodes in the original network in advance (this node has no connecting edges with other nodes), and for this reason, a new inter-regional industrial network of the YRD can be obtained $G_{2012}^{\prime }$ and $G_{2017}^{\prime }$, and the basic information of the network is shown in Table 6.

**Table 6:** Basic information of inter-regional industrial network in Yangtze River Delta based on link prediction.

Network	Number of nodes	Number of edges	Theoretically maximum possible number of edges	Number of known links	Number of unknown links
$G_{2012}^{\prime }$	168	902	14028	902	13126
$G_{2017}^{\prime }$	165	923	13530	923	12607

### Network dataset segmentation

Based on the 2012 and 2017 YRD inter-regional industrial network $G_{2017}^{\prime }\left(V_{2017}^{\prime },L_{2017}^{\prime }\right)$ and $G_{2017}^{\prime }\left(V_{2017}^{\prime },L_{2017}^{\prime }\right)$, a proportion is set to randomly draw edges $L_{2012}^{\prime }$ and $L_{2017}^{\prime }$ in the network into a training set (*proportion*) and a test set (1 − *proportion*), in order to be able to choose a more suitable proportion, the algorithm prediction results will be compared with different proportions. Evaluation of the indicators to choose the AUC, in the AUC evaluation process, random sampling selected 100 independent experiments for comparison. In this paper, we choose 0.4 to 0.9 for the proportion, and make the prediction for it respectively, which is used to verify the relationship between each algorithm and the appropriate proportion matching.

**Fig 2:**
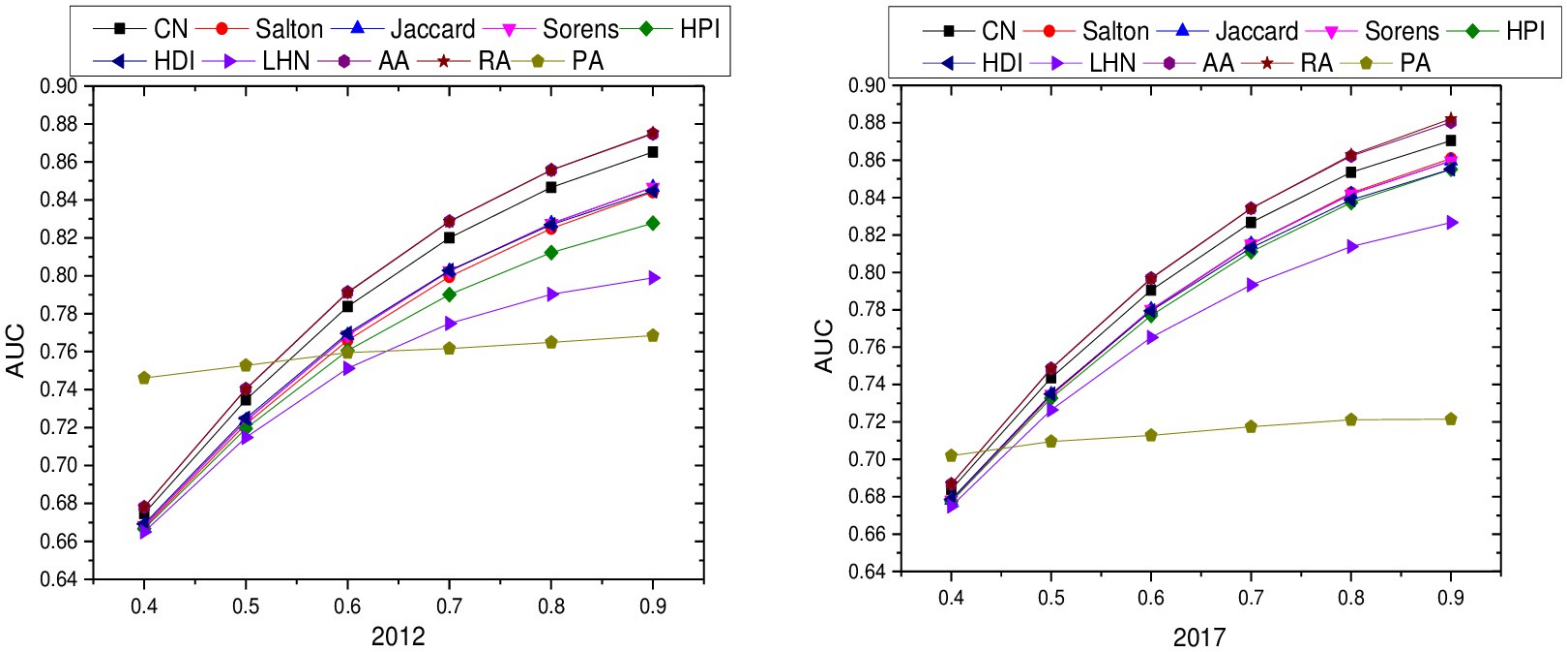
Results of local information similarity indicators with different proportion values in industrial networks in Yangtze River Delta region.

**Fig 3:**
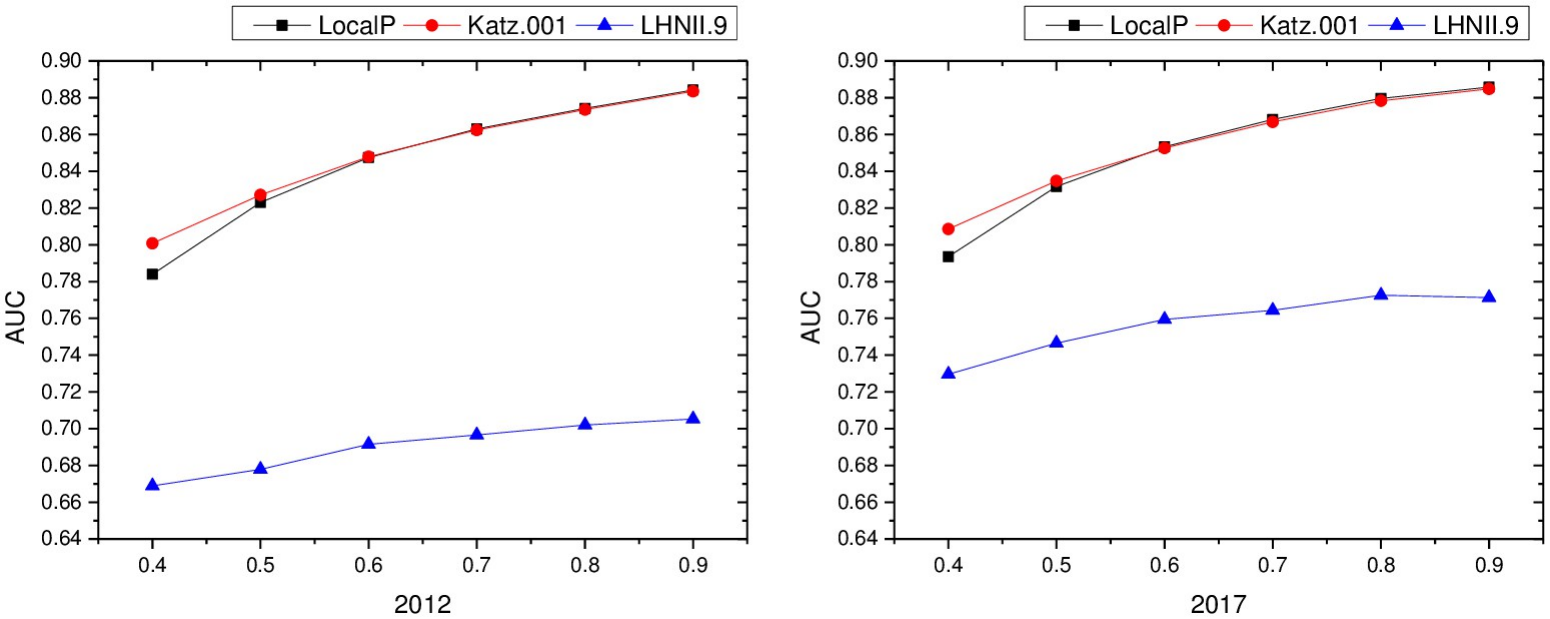
Results of path similarity indicators with different proportion values in industrial networks in Yangtze River Delta region.

**Fig 4:**
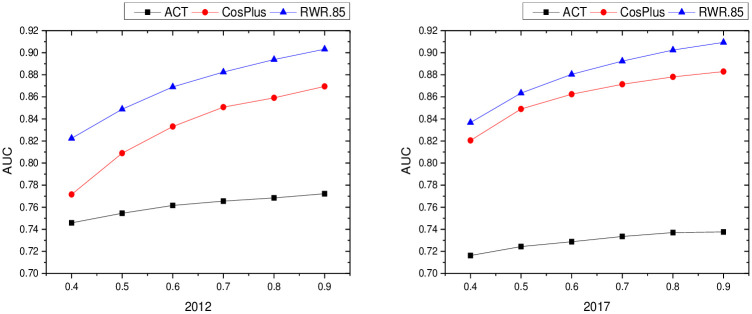
Results of random walk similarity indicators with different proportion values in industrial networks in Yangtze River Delta region.

From Figs 2–4, it can be seen that: different PROPORTION values in the inter-regional industrial network of the YRD in 2012 and 2017 changed differently with the similarity indicators: (1) the PA indicators of local information similarity were insensitive to the PROPORTION value in both 2012 and 2017, and the AUC value did not change much; the other similarity indicators changed gradually with the proportional value changes gradually increase. (2) The AUC value of the LHN-II (0.9) indicator of path similarity in 2012 and 2017 does not change much with different values of proportion. The AUC values of the LP indicator and Katz (0.001) indicator differ very little when the PROPORTION value is taken greater than and equal to 0.6, and the AUC value increases as the PROPORTION value is taken greater. (3) The Cos+ indicator and the RWR indicator of the similarity of the 2012 and 2017 random walks are more sensitive as the proportion takes a larger value.

To summarize, the indicators of local information similarity, path similarity and random wandering similarity in the YRD regional industrial network in 2012 and 2017 increase with the increase of proportion, in other words, the larger the algorithm, the more information the algorithm has, and the better the algorithm’s prediction effect. Therefore, in this paper, we set the value of proportion as 0.9, the test proportion as 10%, and the training set as 90%, and make a one-time division between the test set and the training set to ensure the connectivity of the training set, and pave the way for the subsequent prediction network.

### Predictive modeling

In this paper, we take the 2012 and 2017 YRD inter-regional industrial network ($G_{2012}^{\prime }$,$G_{2017}^{\prime }$) as the base network to construct a link prediction model based on the similarity of the network structure. The essence of the similarity link prediction method based on network structure is an unsupervised sorting method, which scores all node pairs in the network according to the definition of the similarity index, and if the scores of the node pairs in the non-existent edge set(*U*′^*o*^ = *U*′ − *L*′) are all lower than that of the nodes that actually have a connection, we consider that this definition of the similarity index is accurate, and that it is able to correctly predict the links of the network with a greater likelihood.

The process of interregional industrial network link prediction in the YRD in 2012 and 2017 is described as follows:

1. The set of connected edges *L*′ that already exists in the inter-regional industrial network *G*′ is divided into the test set (*L*′^*P*^) and the training set (*L*′^*T*^) respectively by random sampling method.
2. Based on the topology information of the training set (*L*′^*T*^), sixteen similarity matrices are calculated respectively.
3. Using the similarity matrix in step (2), the similarity scores of *L*′^*O*^ and *L*′^*P*^ in the two networks *G*′ are computed separately.
4. Each time a pair of nodes is selected from the test set (*L*′^*P*^), and then a pair of nodes is randomly selected from W. If the score of the former is greater than that of the latter, 1 point is added; if the two are equal, 0.5 point is added.
5. Repeat steps (1) to (4) a finite number of times 100 and take the average of the prediction accuracies of 100 times as the final prediction accuracy of the prediction method.

### Selection of predictive indicators

#### Preliminary selection of predictive indicators

**Fig 5:**
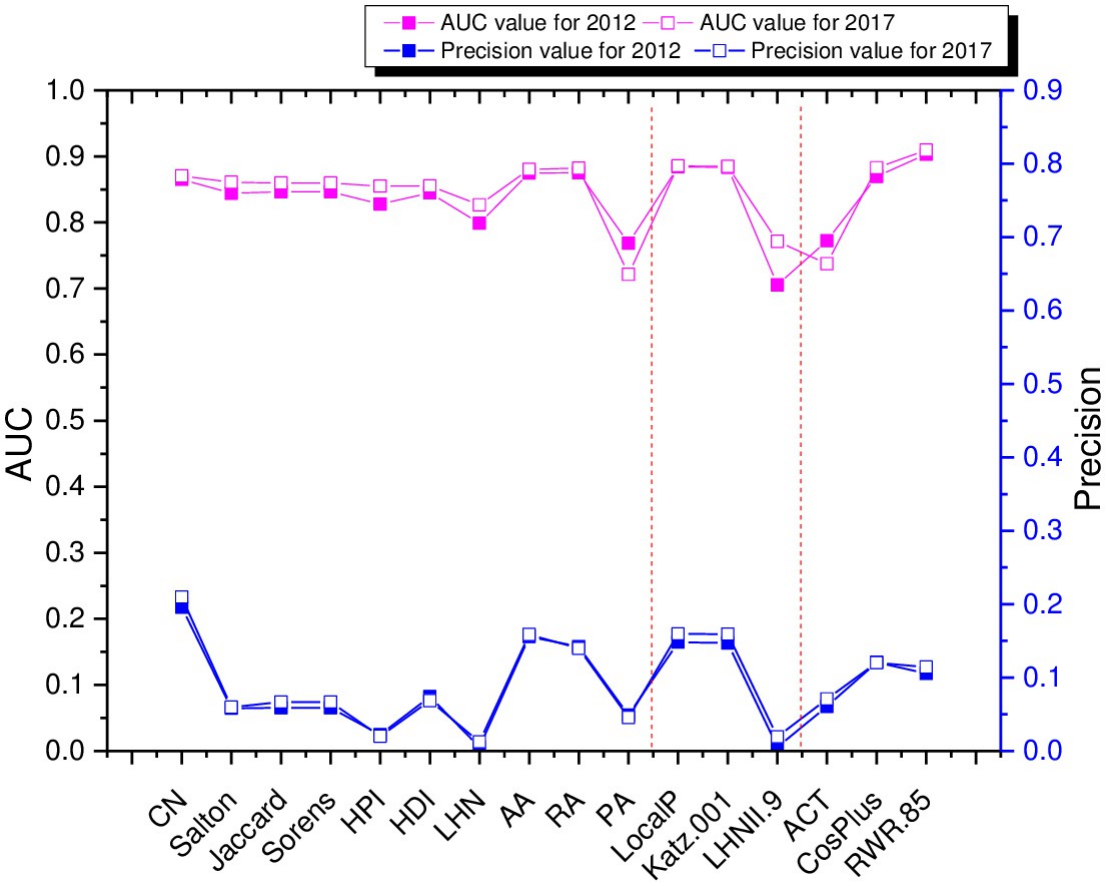
Trend chart of link prediction for industrial network datasets in the Yangtze River Delta region.

Sixteen prediction algorithms in three categories of local information similarity, path similarity and random wandering similarity are selected to evaluate the effect of the prediction model of the industrial network in the YRD region in terms of AUC value and Precision value, which are shown in Fig 5 and Table 7, and the results are as follows:

**Table 7:** Link prediction results for industrial network dataset in Yangtze River Delta region.

		2012		2017	
		AUC	Pre	AUC	Pre
**Local information similarity**	CN	0.8652	**0.1958**	0.8705	**0.2098**
	Salton	0.8442	0.0582	0.8609	0.0598
	Jaccard	0.8466	0.0587	0.8596	0.0667
	Sorens	0.8466	0.0587	0.8596	0.0667
	HPI	0.8277	0.0221	0.8551	0.0200
	HDI	0.8447	0.0738	0.8552	0.0685
	LHN	0.7989	0.0053	0.8267	0.0123
	AA	0.8748	0.1558	0.8803	0.1586
	RA	**0.8752**	0.1420	**0.8821**	0.1397
	PA	0.7685	0.0487	0.7215	0.0455
**Path similarity**	LocalP	**0.8842**	**0.1481**	**0.8858**	**0.1595**
	Katz.001	0.8835	0.1470	0.8848	0.1592
	LHNII.9	0.7053	0.0052	0.7713	0.0192
**Random walk similarity**	ACT	0.7722	0.0604	0.7376	0.0708
	CosPlus	0.8695	**0.1208**	0.8829	**0.1202**
	RWR.85	**0.9032**	0.1055	**0.9094**	0.1141

The predictive effect of the predictive indicators of the industrial network in the YRD region was better in 2012 and 2017, with the same trend of changes in the AUC values of the local information similarity indicator, path similarity indicator (except for the LHNII indicator), and random wandering similarity indicator in the two years.

From the above Table 7, it can be seen that (1) the prediction effect of RA indicator and AA indicator of local information similarity in 2012 and 2017 is much higher than the other indicators, and the AUC values of these two indicators are very close to each other, but the Precision value of RA indicator is higher than that of PA indicator. (2) The predictive effects of LP indicator and Katz (0.001) indicator of path similarity in 2012 and 2017 are very little different, but the Precision value of LP indicator is higher than Katz indicator. (3) The AUC value of the RWR (0.85) indicator of randomized wandering similarity in 2012 and 2017 is much higher than the AUC values of the other indicators, and its Precision value is relatively high. (4) In summary, it can be seen that the RA indicator, the LP indicator and the RWR indicator are selected as the optimal indicators for the algorithms of local information similarity, path similarity and random wandering similarity, respectively.

#### Algorithm validity check

In this paper, RA value, LP value and RWR value are found to have better prediction effect in local information, path and random wandering algorithms, respectively, and produce the highest scores in the industrial networks in the YRD region. In this section, the RA algorithm, LP algorithm and RWR algorithm are used to calculate the likelihood of the existence of a relationship between the test set and nonexistent links in the industrial network among three provinces and one city, and the nonexistent links are ranked according to the RA, LP and RWR values, where the links with a higher value of the indicator indicate a greater likelihood of converting potential inter-industry supply-demand relationships into the establishment of an inter-industry supply-demand relationship. Table 8 shows the top 30 industry pairs with the highest RA value scores in the 2012 YRD regional industrial network forecast (with the connecting edges of the test set excluded), and in order to further analyze whether these potential links will lead to future supply-demand relationships, the top 30 potential relationships are selected with the results of the RA algorithm.

**Table 8:** Top 30 potential links with RA value in the 2012 Yangtze River Delta regional industrial network forecast.

Rankings	Provinces	Industry	Provinces	Industry	RA
**1**	Shanghai	Transportation, storage and postal services	Shanghai	Chemical products	2.2224
**2**	Anhui	Wholesale and retail	Anhui	Chemical products	2.0415
**3**	Zhejiang	Metal smelting and rolling products	Zhejiang	Chemical products	1.7065
**4**	Anhui	Wholesale and retail	Anhui	Metal smelting and rolling products	1.6262
**5**	Shanghai	Transportation, storage and postal services	Shanghai	Metal smelting and rolling products	1.3457
**6**	Zhejiang	Chemical products	Zhejiang	Paper, printing and educational and sporting goods	1.1190
**7**	Shanghai	Financial	Shanghai	Chemical products	0.9704
**8**	Shanghai	Metal smelting and rolling products	Shanghai	Chemical products	0.9457
**9**	Anhui	Leasing and business services	Jiangsu	Leasing and business services	0.9421
**10**	Anhui	Leasing and business services	Anhui	Real estates	0.9303
**11**	Anhui	Transportation, storage and postal services	Anhui i	Production and supply of electricity and heat	0.9090
**12**	Anhui	Metal smelting and rolling products	Anhui	Chemical products	0.8679
**13**	Anhui	Production and supply of electricity and heat	Shanghai	Financial	0.8646
**14**	Zhejiang	Chemical products	Shanghai	Financial	0.8622
**15**	Anhui	Production and supply of electricity and heat	Zhejiang	Financial	0.8438
**16**	Zhejiang	Financial	Zhejiang	Production and supply of electricity and heat	0.8317
**17**	Shanghai	Constructions	Shanghai	Chemical products	0.7937
**18**	Anhui	Leasing and business services	Anhui	Transportation, storage and postal services	0.7756
**19**	Zhejiang	Transportation, storage and postal services	Zhejiang	Chemical products	0.7552
**20**	Zhejiang	Transportation, storage and postal services	Zhejiang	Chemical products	0.7552
**21**	Anhui	Real estates	Anhui	Metal Ore Mining Products	0.7515
**22**	Anhui	Wholesale and retail	Anhui	Constructions	0.7435
**23**	Zhejiang	Leasing and business services	Zhejiang	Chemical products	0.7351
**24**	Shanghai	Real estates	Shanghai	Transportation, storage and postal services	0.7283
**25**	Shanghai	Transportation, storage and postal services	Shanghai	Wholesale and retail	0.7196
**26**	Zhejiang	Leasing and business services	Zhejiang	Metal smelting and rolling products	0.7101
**27**	Anhui	Leasing and business services	Anhui	Chemical products	0.6925
**28**	Anhui	Leasing and business services	Anhui	Metal smelting and rolling products	0.6908
**29**	Jiangsu	Chemical products	Jiangsu	Agricultural, forestry and fishery products and services	0.6792
**30**	Anhui	Constructions	Anhui	Metal Ore Mining Products	0.6744

**Fig 6:**
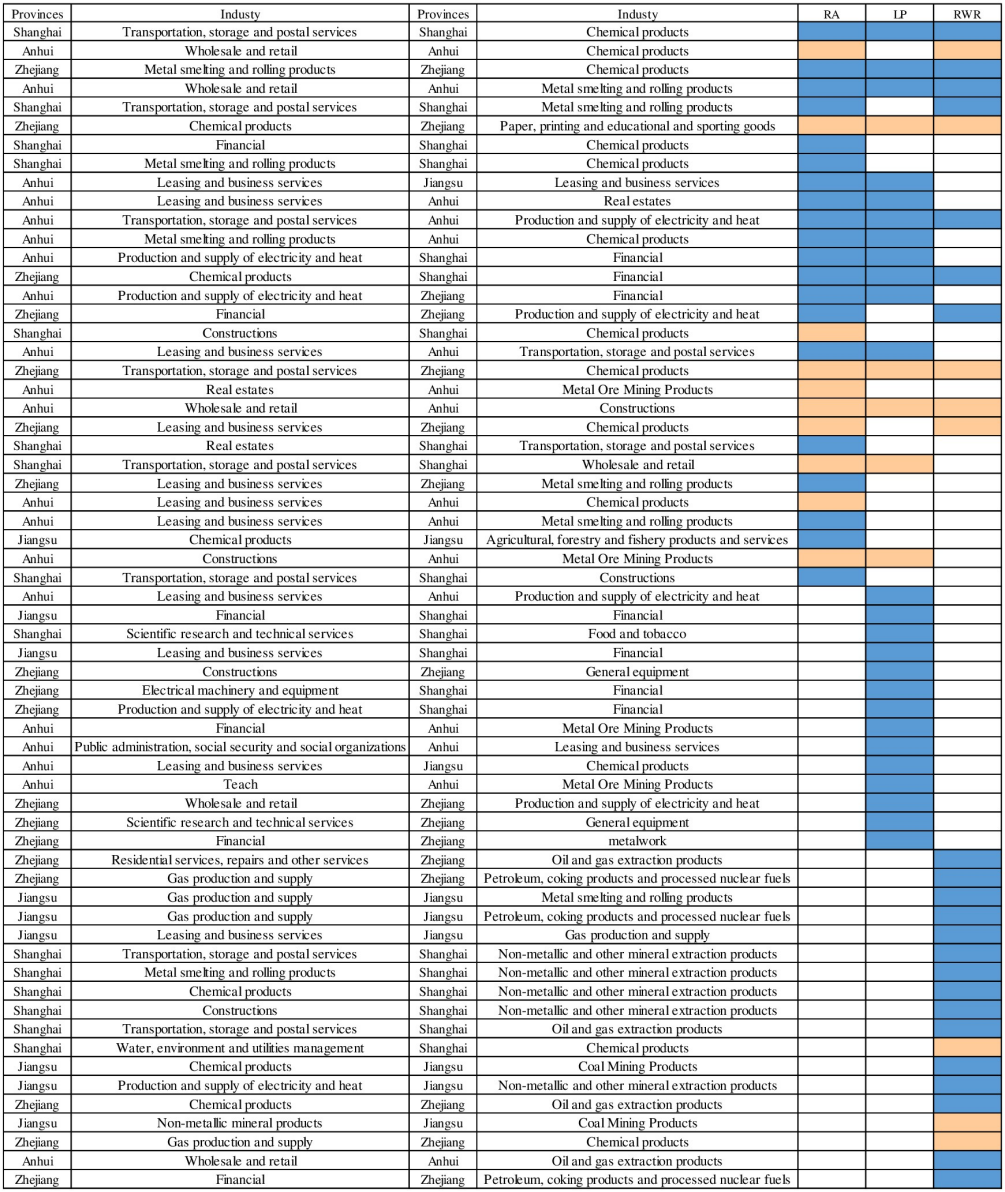
Potential industrial relationships in the industrial network of the Yangtze River Delta region.

The top 30 industry pairs were selected for each of the RA, LP and RWR indicators, resulting in a total of 90 industry-demand-supply pairs. However, due to the fact that some "potential supply-demand relationships" are repeated in the top 30 in different indicators, a total of 63 pairs of industry pairs are obtained by eliminating the duplicated pairs. In order to investigate the effectiveness of RA, LP and RWR algorithms, and to find the potential patterns of supply and demand relationships between industries in the YRD region, we select the pairs that do not have links among the top 30 pairs in 2012 in terms of RA, LP and RWR values, and define them as "potential supply-demand relationships", and observe whether these pairs have established supply-demand relationships in 2017. The results of the comparison are shown in Fig 6. The blue squares in each row indicate that the industrial pair did not actually have an industrial supply-demand relationship in 2017, but the value of an algorithmic indicator for this pair of links scored in the top 30, so the study predicted them as potential supply-demand relationships. Orange squares indicate that the industry pair actually conducted industrial supply and demand relationships in 2017, and they are links that actually exist in real industrial supply and demand relationships. If in a row, there are orange squares, it means that the industrial supply and demand links of that industrial pair successfully occurred after applying a certain algorithmic indicator prediction, the potential link of that pair is a valid prediction; on the contrary, it is an invalid prediction.

By comparing the forecasting results of RA indicator, LP indicator and RWR indicator, it can be found that 10 out of the first 30 industry pairs predicted by RA indicator were successfully predicted, which is higher than the prediction success rate of the other two indicators, which indicates that RA algorithm has certain effectiveness and accuracy compared with LP algorithm and RWR algorithm in predicting the potential relationship between industries in the industrial network of the YRD region. and accuracy, and is a better algorithm to predict the potential future supply and demand relationships between industries in the YRD region.

### Network evolution prediction results

**Fig 7:**
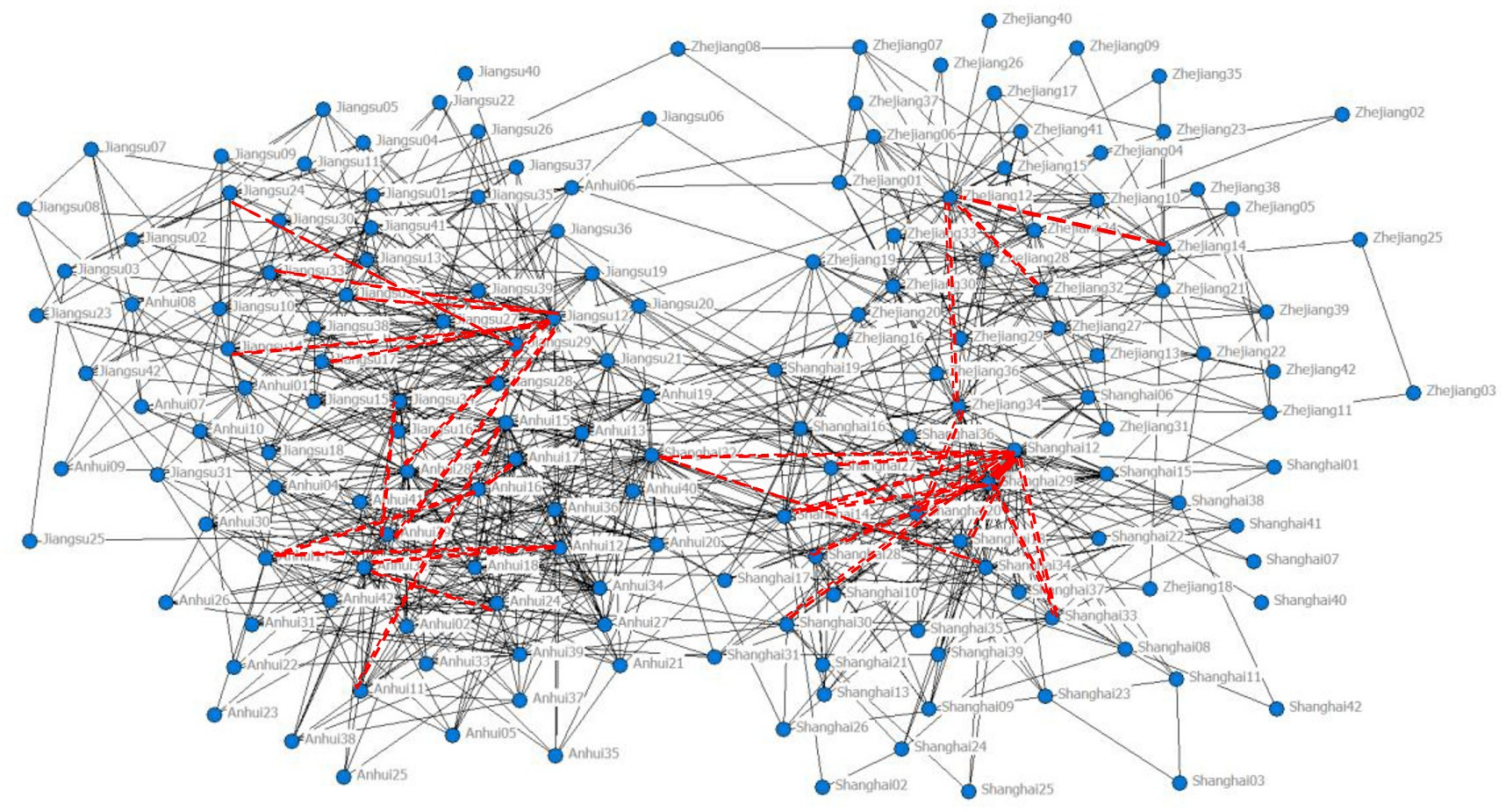
Forecast of future links in the Yangtze River Delta regional industrial network.

Due to the lag in the preparation and release of input-output tables, in order to meet the uniformity and availability of data caliber, the most recent 2017 YRD inter-regional industrial network $G_{2017}^{\prime }$ was selected as the link prediction base network, the RWR index of random walk similarity is used to calculate the network structure similarity and to predict the inter-regional industrial network structure, so as to determine whether an industrial node in the YRD region has a cooperative relationship with another industrial node. With the help of MATLAB software, the RA value of potential industrial pairs in the industrial network of the YRD region (excluding the test set of connected edges) is calculated, in which the larger the RA value of each potential industrial pair is, the higher the possibility of the existence of links between them. In this paper, we take the first 30 pairs of industrial pairs by combining the S values of the industrial networks in the YRD region that have not yet been connected to the edges. In the future development of industries in the YRD region, the top 30 pairs of industry pairs are most likely to generate supply and demand relationships, of which the top 30 pairs of industry pairs are shown in Table 9, and the future connected edges of these 30 pairs of industries are shown in Fig 7.

**Table 9:** Predicting future link results of industrial networks in the Yangtze River Delta region based on RA indicators (Top 30 pairs).

Provinces	Industry	Provinces	Industry	RA
Shanghai	Wholesale and retail	Shanghai	Chemical products	3.3557
Zhejiang	Metal smelting and rolling products	Zhejiang	Chemical products	1.7314
Jiangsu	Metal smelting and rolling products	Jiangsu	Chemical products	1.2828
Anhui	Information transmission, software and information technology services	Anhui	Chemical products	1.2305
Shanghai	Wholesale and retail	Shanghai	Constructions	1.1692
Shanghai	Transportation, storage and postal services	Shanghai	Chemical products	1.0232
Shanghai	Financial	Shanghai	Wholesale and retail	0.9823
Jiangsu	Specialized equipment	Jiangsu	Chemical products	0.9616
Shanghai	Wholesale and retail	Shanghai	Metal smelting and rolling products	0.9564
Jiangsu	Information transmission, software and information technology services	Jiangsu	Chemical products	0.924
Anhui	Metal smelting and rolling products	Anhui	Chemical products	0.9137
Shanghai	Metal smelting and rolling products	Shanghai	Chemical products	0.9064
Anhui	General equipment	Anhui	Metal smelting and rolling products	0.8897
Anhui	Constructions	Jiangsu	Chemical products	0.879
Anhui	Information transmission, software and information technology services	Anhui	Metalwork, machinery and equipment repair services	0.8779
Anhui	Wholesale and retail	Jiangsu	Chemical products	0.8457
Jiangsu	Wholesale and retail	Jiangsu	Metalwork, machinery and equipment repair services	0.8418
Zhejiang	Real estates	Zhejiang	Chemical products	0.8043
Shanghai	Water production and supply	Shanghai	Chemical products	0.801
Anhui	Specialized equipment	Anhui	General equipment	0.7881
Zhejiang	Real estates	Shanghai	Communications equipment, computers and other electronic equipment	0.776
Shanghai	Real estates	Shanghai	Information transmission, software and information technology services	0.7714
Shanghai	Communications equipment, computers and other electronic equipment	Shanghai	Chemical products	0.7705
Shanghai	Transportation equipment	Shanghai	Chemical products	0.765
Jiangsu	Financial	Jiangsu	Chemical products	0.7528
Anhui	Wholesale and retail	Jiangsu	Real estates	0.7406
Zhejiang	Information transmission, software and information technology services	Zhejiang	Chemical products	0.7401
Shanghai	Information transmission, software and information technology services	Shanghai	Chemical products	0.736
Shanghai	Financial	Shanghai	Chemical products	0.7323
Anhui	Metalwork	Anhui	Petroleum, coking products and processed nuclear fuels	0.718
Shanghai	Wholesale and retail	Shanghai	Chemical products	3.3557
Zhejiang	Metal smelting and rolling products	Zhejiang	Chemical products	1.7314
Jiangsu	Metal smelting and rolling products	Jiangsu	Chemical products	1.2828
Anhui	Information transmission, software and information technology services	Anhui	Chemical products	1.2305
Shanghai	Wholesale and retail	Shanghai	Constructions	1.1692
Shanghai	Transportation, storage and postal services	Shanghai	Chemical products	1.0232
Shanghai	Financial	Shanghai	Wholesale and retail	0.9823
Jiangsu	Specialized equipment	Jiangsu	Chemical products	0.9616

**Note:** The solid line in the figure indicates the existing connecting edges and the dashed line indicates the most likely potential connecting edges in the future.

As can be seen from Table 9 and Fig 7 above, (1) in the prediction results of future links in the first 30 pairs of industry pairs in the YRD region, the industries of each province and city have more opportunities for cooperation within their own provinces and municipalities, and there are fewer opportunities for industrial cooperation among the three provinces and one city. To realize the development of YRD regional integration, especially the development of industrial integration, the joint efforts of all parties in the region are needed. (2) There are relatively few opportunities for inter-industry supply cooperation among the three provinces and one city, including “Construction (28)” in Anhui Province and “Chemical products (12)” in Jiangsu Province, “Wholesale and retail (29)” in Anhui Province and "Chemical products (30)" in Jiangsu Province, and “Wholesale and retail (31)” in Anhui Province. “Chemical products (12)” in Anhui Province and “Real estate (34)” in Zhejiang Province and “Communication equipment, computers and other electronic equipment (20)” in Shanghai between more opportunities for cooperation in the future. (3) The RA values between “Wholesale and retail (29)” and "Chemical products (12)” in Shanghai, “Metal smelting and rolling products (14)” and "Chemical products (12)” in Zhejiang Province, “Metal smelting and rolling products (14)” and “Chemical products (12)” in Jiangsu Province and “information transmission, software and information technology services (32)” and “chemical products (12)” in Anhui Province are 3.3557, 1.7314, 1.2828 and 1.2305, and these industries have greater opportunities for cooperation in the future. The YRD region is not only the leading economically developed region in China, but also the most developed and concentrated region of chemical industry in China, with the layout and development level of chemical parks and the management level ranking among the top in the country. In order to further promote the rapid development of chemical industry in the YRD, it is necessary to continuously extend and broaden the chemical industry chain, so that more industries and the chemical industry to generate supply relationships and improve the chemical industry chain. (4) “Chemical products (12)”, “Construction (28)”, “Information transmission, software and information technology services (32)” and “Real estate (34)” in the three provinces and one city are in an important position in the future cross-regional industrial network and have the information advantage and control advantage.

## Countermeasures and recommendations

Through the above conclusions, it is not difficult to find that the industrial homogeneity among the provinces and cities in the YRD region is relatively strong, leading to homogeneous competition and difficulties in industrial division of labor; at the same time, the nature of the YRD industrial layout co-ordination is not prominent, and the regional industrial layout focuses on the provinces and municipalities themselves, lacks the thinking of cross-regional layout, and lacks the depth and strength of the cross-regional co-operation in the industry, and so on. As the YRD region is one of the regions with the most active economic development, the highest degree of development and the strongest innovation ability in China, and has a pivotal strategic position in the overall situation of national modernization and all-round opening pattern, how to reasonably plan the industrial layout and vigorously rationalize the industrial chain in accordance with the overall functional positioning of the YRD region and the specific functional positioning of three provinces and one city, so as to form a rational industrial layout and a linkage mechanism for upstream and downstream industrial development among three provinces and one city is an urgent and important solution at this stage. It is an important problem to be solved at this stage to build the industrial network of the YRD region into a highland of industrial development with complete structure, significant core, outstanding comparative advantages and obvious interconnectivity.

### From the government’s macro perspective

#### Seeking innovative governance models to enhance cross-regional collaboration

The YRD region covers a wide range of areas and is a cross-provincial and municipal administrative region, making it more difficult to form a unified and effective inter-regional collaborative governance mechanism and cooperation mechanism. Continuously searching for and innovating cross-regional communication platforms at the level of provincial and municipal governments as well as common governance models, and strengthening the construction of cross-regional inter-regional cooperation mechanisms are the first and most difficult tasks in the industrial integration of the YRD. Insist on seeking reform programs suitable for the moderate separation of economic zones and administrative regions, and promote synergistic development among provinces and municipalities within the YRD region.

Priority has been given to promoting an "appropriate" division and merger of administrative competencies among provinces and municipalities, and improving the mechanism for cross-regional coordination and cooperation. On the basis of the existing series of cooperation agreements on the construction of interregional economic zones, the administrative authorities of provinces and municipalities should be further consolidated. The municipal governments of the three provinces and one city will jointly set up an inter-provincial and municipal coordinated development leading group that overrides administrative divisions to coordinate the management of economic development competencies, and set up under it deliberative and arbitration bodies, joint meetings and joint work-promotion groups for the interconnected construction of transportation and the matching and coordination of elemental resources, so as to promote localized regional co-citizenship. At the same time, the provinces and cities in the region cede their economic management authority, retain the right to share tax revenue and social management authority, focus on building cross-regional public service integration, and adopt “one area with multiple parks”, “enclave building parks”, “joint investment promotion” and other inter-regional cooperation modes, seize the opportunity of inter-regional cooperation platform construction, analyze the comparative advantages of each province and city, and participate in the construction of inter-regional industrial chains, so that the region as a whole can maximize its benefits.

#### Coordination of cross-regional interests and strengthening of cross-regional synergistic mechanisms

As there are differences in the level of development and interests of the three provinces and one city in the YRD, it is inevitable that the three provinces and one city government will have incomplete communication and access to information, rupture of trust, and even breach of agreement, failure to fulfill their duties, and sabotage of cooperation. Due to the imperfection of the cross-regional cost-sharing mechanism and benefit-sharing mechanism, even if the concepts and goals are agreed upon, the party involved in the cooperation will be subjected to the pressure of larger institutional transaction costs and economic transformation costs, and the cross-regional cooperation will be difficult. For this reason, to determine the interests of the three provinces and one city, it is necessary to improve the cross-regional interest coordination mechanism. Cross-regional interest coordination mechanisms mainly include mechanisms for expression and demand, consultation and agreement, sharing and compensation, supervision, dispute resolution, dynamic evaluation and feedback.

#### Sound hardware and software facilities to optimize the environment for sustainable development

Adopting the integration strategy of “connectivity, sharing and collaboration” to further deepen the integration of social resources and public services in the YRD region. The integration of social resources and public services will assist in the overall development of the cross-regional economy, match the process of cross-regional economic integration, promote the high-quality development of the YRD region’s economy, ensure the free flow of talents in the YRD region, and enable residents in the YRD region to enjoy high-quality public services without barriers, high efficiency or discrimination.

The integration of infrastructure, especially transportation facilities, can improve cross-regional accessibility. Specifically, it is necessary to increase investment in transportation construction in the YRD region, focusing on strengthening the construction of highways, railroads, and passenger and freight transportation networks between major cities in the region and continuously upgrading the capacity of the sections that are now open to traffic, so as to satisfy the future demand for the flow of factors of production and talents among industries across the region; strengthening the construction of transportation channels to the sea in important coastal ports in the YRD region, so as to promote a mesh structure of It is necessary to strengthen the construction of sea transportation corridors for important coastal ports in the YRD region, so that cities in the YRD region will have a net-like structure of transportation routes, and to ensure the autonomy and diversity of cross-regional industrial development and factor flows; it is also necessary to strengthen the establishment of a ring-shaped rapid transportation network around major large cities, with rail transportation as the main focus, so as to facilitate the adjustment and upgrading of the cross-regional industrial structure and the expansion of the economic frontier between the core cities in the region and the surrounding peripheral areas.

### From the industry’s meso perspective

#### Precise planning of industrial staggered development, to avoid non-order competition

In the process of industrial development in the YRD region, it is necessary to focus on the leading and driving role of the advantaged industries in the region to their counterparts as well as their radiating role to other industries and other regions in the region. (1) Shanghai should strengthen the resource allocation function of the international financial center, actively explore qualified offshore investment, fully participate in the cooperation of Shanghai’s various types of factor market allocation, vigorously attract the headquarters of international financial organizations and domestic and foreign large-scale financial institutions to move in, establish a global asset management center, and accelerate the creation of a global RMB product center, trading, pricing and clearing center. Enhance its role as a hub in the global trade network, vigorously promote the innovative development of cultural transactions, technology trade, re-export trade and offshore trade, create a number of large markets and platforms serving the integration of the YRD region, facing the whole country, facing the international arena, connecting internal and external, and linking up futures and cash, and accelerate the development of cross-border e-commerce, digital trade, and other new types of trade business. (2) Jiangsu Province should focus on promoting the development of high-quality manufacturing industry, actively participate in the system and implementation of the YRD Manufacturing Synergistic Development Plan, study the policies and measures to refine the advantageous manufacturing industry, and work hard to enhance the advantages of competition and cooperation. (3) Zhejiang Province needs to accelerate the development of modern service industry and create a new engine of modern service industry in the YRD. Focusing on new business forms such as platform economy, sharing economy, experience economy and creative economy in the service industry, it should enhance the e-commerce application platform, build an integrated capacity sharing platform, cultivate a new mode of experience economy, and promote the integration of creative economy and traditional economy. (4) Anhui Province should focus on developing digital industrialization and industrial digitization and vigorously promote digital empowerment, while using PR management, social security and social organizations to improve the industry’s own strengths and enhance its ability to transfer industries.

#### Extending cross-regional industrial chains and promoting the formation of cross-regional industrial clusters

The extension of the transregional industrial chain upstream is the key to ensuring the safety and stability of the transregional industrial chain. The upstream of the cross-regional industrial chain is the starting end of the entire industrial chain, providing industries for the production and manufacturing of raw materials and components. Among them, the upstream industries often hold certain resources, such as key basic materials and core technologies, and have strong core competitiveness, which is the weak link in the cross-regional industrial chain. Extending to the upstream of the cross-regional industrial chain can make up for the shortcomings of the cross-regional industrial chain, establish an autonomous, controllable, safe and reliable production and supply system in the region, and enhance the integrity, stability and competitiveness of the cross-regional industrial chain.

Extending the cross-regional industrial chain downstream is an important initiative to provide industrial added value. In the cross-regional industrial chain, industrial added value is more manifested in R&D and market. Extending to the downstream of the cross-regional industrial chain, entering the market expansion link, accurately matching the new consumption and market demand, promoting the application with innovation, promoting the development with application, and obtaining profits from the market. Enhancing industrial value-added through deep processing, advancing to the middle and high end of the value chain, and lengthening the cross-regional industrial chain will help consolidate the stock and enlarge the incremental market.

The extension of cross-regional industrial chains is conducive to the formation of cross-regional industrial clusters. Based on the advantageous industries in the region, it promotes the extension of the cross-regional industrial chain to the upstream and downstream, builds a complete cross-regional industrial chain, and brings into play the effect of cross-regional industrial agglomeration. This can not only improve the overall competitive advantage of cross-regional industries, but also utilize the comparative advantage of location to provide supporting sharing of products, technologies, services and human capital, thus realizing a balanced cross-regional industrial division of labor and competition.

#### Enhancing the integration of cross-regional industrial and innovation chains to achieve high-quality cross-regional economic development

Through the integration of industrial chain and innovation chain, adjust the layout of strategic emerging industries and future industrial development. First, vigorously develop the digital economy. Focusing on the weak links in the industrial chain of the YRD region, we will overcome key technologies and build new infrastructures, encourage the application of digital technologies by Internet platform enterprises to help the transformation and upgrading of the real economy, support the extension of leading enterprises in various industries to the upstream and downstream of the cross-regional industrial chain through mature Internet platforms, improve the digitization of the whole industry, give birth to new industries, new forms and new models, expand new space for development, and cultivate new kinetic energies, so as to better improve the production efficiency and enhance the development of new industries. dynamic energy, and better improve productivity, economic efficiency and high-quality development. Secondly, cultivate and grow intellectual property-intensive industries. Focusing on strategic emerging industries such as new materials, medicine and healthcare, new equipment, etc., it develops intellectual property financial services, rapidly transforms and applies intellectual property achievements, builds public service platforms for intellectual property-intensive industries, and accelerates the development of knowledge industry-intensive industries. At the same time, strengthen the scientific and technological research support for intellectual property-intensive industries, improve the tolerance of their scientific and technological research and innovation failures, increase the support for their exemplary applications, create an early market for exploratory products, encourage leading users to try them out, and promote technological innovation in knowledge industry-intensive industries. Third, optimizing the layout of future industrial development. There is an urgent need to optimize the layout of future industrial development in the YRD on the basis of cutting-edge science and technology and the direction of key core technology changes and innovations, and with the guarantee of building new policies on cutting-edge technology incubation, diversified inputs, early market cultivation and the creation of industrial ecosystems, and with the orientation of "technology generates demand and demand leads the industry", to optimize the layout of future industrial development in the YRD. In order to optimize the layout of future industry development in YRD, we will promote the future industry to become a fresh force for the high-quality development of cross-regional economy.

Through the integration of cross-regional industrial chains and innovation chains, the shortcomings and pain points of cross-regional industrial chains will be remedied and eliminated. Increase regional investment in "hard science and technology" innovation, set up more scientific and technological research projects for the main battlefield of the cross-regional economy, explore cross-regional national laboratories and other new research and development institutions, and focus on key core technologies from the perspective of the YRD region’s urgent and long-term needs, insisting on independent innovation, persistent research and achieving major breakthroughs. Through tax incentives and other measures to mobilize the enthusiasm of enterprises in independent research and development, and deepen the market allocation of key core technological innovation factors; through the "horse racing" and "list of commanders" systems, promote the docking of the industrial chain and the innovation chain, and promote the development of technological innovation and scientific and technological achievements of scientific research institutes in accordance with industrial demand. Through the systems of "horse racing" and "ranking the leaders", promote the docking of the industrial chain with the innovation chain, promote technological innovation and the transformation of scientific and technological achievements by scientific research institutes based on industrial demands, enhance the scientific and technological supply capacity of the key links in the trans-regional industrial chain, and ensure the security of the trans-regional industrial chain.

#### Integrated planning of strategic industries to enhance cross-regional competitiveness

The development of industries in the YRD region should not only focus on the present, but also look to the future, so as to provide sustained impetus for the economic development and industrial integration of the YRD region. On the one hand, it is necessary to determine the strategic position of core emerging industries in the three provinces and one city, and to coordinate the planning and layout. Cultivating and growing information technology, high-end equipment manufacturing, new materials and other emerging industries is not only the proper meaning of optimizing the economic structure, but also a necessary move to build a modernized system, which will surely stimulate stronger development momentum. At present, the three provinces and one city in the YRD have layouts in these industries, each with its own advantages and disadvantages, the overall industrial correlation is relatively low, and the provinces and cities present a competitive situation. If the three provinces and one city industry for integrated planning, the advantages and disadvantages of integration, the formation of a cross-regional industrial clusters, together with the formation of emerging industries town, the YRD region emerging industries on a new height.

On the other hand, it is necessary to analyze the existing traditional industry profiles of the three provinces and one city to create a cross-regional industrial cluster advantage with special characteristics. Traditional industries (e.g. petrochemical industry, construction industry, etc.) are the bases of the modern industrial system, and are in an indispensable position in the cross-regional industrial chain. At present, the petrochemical industry has too high a degree of external dependence, which is vulnerable to changes in the international political and economic situation, and thus affects economic security and even national security. Therefore, it is necessary to accelerate the integrated planning of the layout of the petrochemical industry in the YRD region, taking into account not only the supply and demand of raw materials and resources, but also the supply and demand of the upstream and downstream of the industrial chain as well as the differentiation of the product structure; at the same time, the YRD region is the region with the highest degree of high-end manufacturing industry concentration, which are the main supporting fields of the new chemical materials, specialty chemicals and high-end composites, and synergistic development of the petrochemical industry in the YRD region and the high-end manufacturing industry The effect of synergistic development of petrochemical industry and high-end manufacturing industry in the YRD region is obvious. Therefore, the YRD region should develop synergistically and plan the industrial layout in a coordinated manner, so as to create a unique and indispensable resource-based and energy-based industrial cluster.
